# Epigallocatechin-3-Gallate, Quercetin, and Kaempferol for Treatment of Parkinson’s Disease Through Prevention of Gut Dysbiosis and Attenuation of Multiple Molecular Mechanisms of Pathogenesis

**DOI:** 10.3390/brainsci15020144

**Published:** 2025-01-31

**Authors:** Alexis Kalu, Swapan K. Ray

**Affiliations:** Department of Pathology, Microbiology and Immunology, University of South Carolina School of Medicine, 6439 Garners Ferry Road, Columbia, SC 29209, USA; alexis.kalu@uscmed.sc.edu

**Keywords:** Parkison’s disease (PD), epigallocatechin-3-gallate, quercetin, kaempferol, gut microbiome, neuroinflammation, mitochondrial dysfunction

## Abstract

Parkinson’s disease (PD) is a neurodegenerative condition in which degeneration mostly occurs in the dopamine (DA)-producing neurons within the substantia nigra in the midbrain. As a result, individuals with this condition suffer from progressively worsening motor impairment because of the resulting DA deficiency, along with an array of other symptoms that, over time, force them into a completely debilitating state. As an age-related disease, PD has only risen in prevalence over the years; thus, an emphasis has recently been placed on discovering a new treatment for this condition that is capable of attenuating its progression. The gut microbiota has become an area of intrigue among PD studies, as research into this topic has shown that imbalances in the gut microbiota (colloquially known as gut dysbiosis) seemingly promote the primary etiologic factors that have been found to be associated with PD and its pathologic progression. With this knowledge, research into PD treatment has begun to expand beyond synthetic pharmaceutical compounds, as a growing emphasis has been placed on studying plant-derived polyphenolic compounds, namely flavonoids, as a new potential therapeutic approach. Due to their capacity to promote a state of homeostasis in the gut microbiota and their long-standing history as powerful medicinal agents, flavonoids have begun to be looked at as promising therapeutic agents capable of attenuating several of the pathologic states seen amidst PD through indirect and direct means. This review article focuses on three flavonoids, specifically epigallocatechin-3-gallate, quercetin, and kaempferol, discussing the mechanisms through which these powerful flavonoids can potentially prevent gut dysbiosis, neuroinflammation, and other molecular mechanisms involved in the pathogenesis and progression of PD, while also exploring their real-world application and how issues of bioavailability and potential drug interactions can be circumvented or exploited.

## 1. Introduction

**Figure 1:**
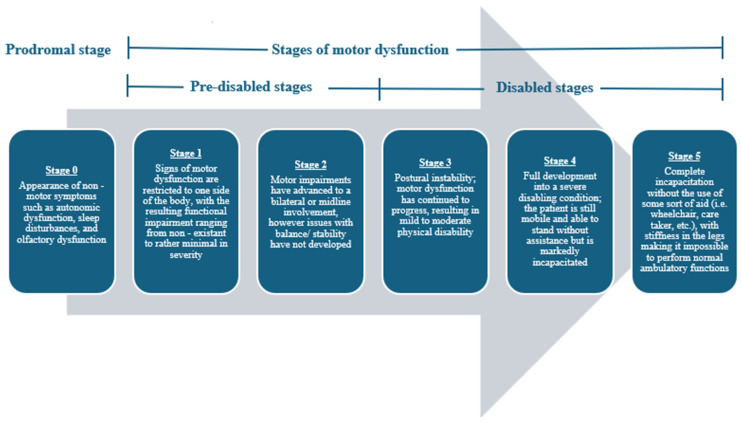
Progression of motor impairment among individuals with PD using the 5-stage scaling system developed by Hoehn et al. [6]. The inclusion of the prodromal stage of stage 0 serves as a slight adaptation to this scaling system, and it is used to represent the non-motor symptoms that often compromise this disease in its earliest presentations.

In 1817, James Parkinson first noted the clinical presentation of Parkinson’s disease (PD) [1]. PD is an age-related neurodegenerative condition clinically characterized by the presence of toxic alpha-synuclein (α-synuclein) aggregates in Lewy body inclusions. Now, PD is regarded as the second most common neurodegenerative disease and the most common movement disorder in the world [2,3]. PD occurrence worldwide has doubled over the past 25 years, and its clinical severity is a leading cause of disability in adults [2,3,4]. At its onset, PD is characterized and diagnosed by an exhibition of resting tremors, bradykinesia (slowness of movement), and rigidity, but as PD progresses, the motor dysfunction only worsens in severity [1,5]. Before its clinical manifestation, PD presages some notable non-motor warning signs such as autonomic nervous system impairments, sleep interruptions, and olfactory system dysfunction [1,4,5]. The clinical progression of PD is often described using a five-stage scaling system (Figure 1) [6,7]. This disease is debilitating; thus, with its mounting prevalence across the globe, both physicians and researchers alike are trying to gain a better understanding of the etiology behind this condition to devise effective physical, pharmacological, and phytochemical therapies.

Degeneration of dopamine (DA)-producing or dopaminergic neurons in the substantia nigra (SN) has long been implicated in the development of PD and the progressive deterioration of motor function. The SN is composed of two regions, the pars compacta and the pars reticularis, and it is from the pars compacta that dopaminergic projections span and synapse upon the striatum, giving way to opposing pathways that directly inhibit and indirectly stimulate the globus pallidus internus, regulating the initiation of motor output [8]. The implications of the degeneration of dopaminergic neurons are well understood; however, research is still being conducted to determine the causative factors that provoke this process. Many investigators currently think that PD is a multifactorial disease, and among these potential factors, genetics, environmental exposure, and increasing age are often revered as the most important components. This being said, the exact cause of PD is still unknown; thus, current treatment strategies only focus on reducing the symptoms of this disease by augmenting the DA pathway.

**Figure 2:**
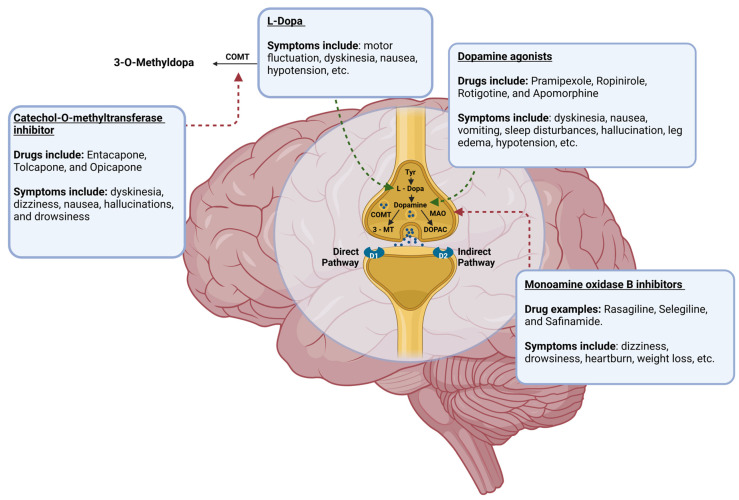
The mechanism of DA biosynthesis and the points at which current pharmaceutical agents act to mediate the alleviation of symptoms of PD through augmentation of the pathway, with examples of pharmaceuticals that fall under each class and the most common side effects seen with their administration included. Tyr = tyrosine; COMT = catechol-O-methyltransferase; MAO = monoamine oxidase A; 3-MT = 3-methoxytyramine; DOPAC = 3,4-dioxyphenylacetic acid. This figure was created using BioRender.

Dopaminergic neurons in the midbrain SN produce DA, an essential neurotransmitter in the central nervous system (CNS). DA levels steadily decline in the CNS due to the degeneration of dopaminergic neurons in PD [9]. So, augmentation of the DA signaling pathway is an objective in the pharmaceutical treatment of PD (Figure 2) [1,4]. Through this mechanism, DA agonists, monoamine oxidase-B inhibitors (MAOBIs), and catechol-O-methyltransferase inhibitors (COMTIs) alleviate motor dysfunctions in PD. However, none of these drug classes are as efficacious as Levodopa (L-Dopa), a gold standard for PD treatment [1,10]. L-Dopa, as a DA precursor, increases DA levels by pushing DA biosynthesis forward, augmenting the DA signaling pathway. Despite its success, the use of this drug has caused apprehension in recent years as L-Dopa may be neurotoxic and paradoxically quickens the progression of PD instead of its inhibition [4,5,10,11]. While some studies suggest that L-Dopa may be neurotoxic, other studies show that the progression of neurodegeneration is no different in PD patients who receive L-Dopa [10,11]. This debate is ongoing; however, the motor impairments that have been seen with the use of L-Dopa are beyond dispute. Prolonged L-Dopa use induces severe motor fluctuations and dyskinesia (involuntary, erratic, writhing movements) in individuals with PD [1,4,5,10,11]. In addition to this, the use of DA agonists can result in hypotension, leg edema, hallucinations, and sleep disturbances, while deep brain stimulation can lead to worsening dyskinesia, mild cognitive impairment, gait abnormalities, and surgical complications [1,4,12]. An array of side effects can be seen with the use of these drugs, and their utility in the treatment of PD is limited to the management of motor symptoms only; thus, the exploration of other preclinical and clinical treatments is essential if a true treatment for curing PD is to ever be found.

Some recent reports show that much like Alzheimer’s disease (AD) and other neurodegenerative conditions, PD results from neuroinflammation via the gut–brain axis (GBA), oxidative stress, and dysfunctions in the ubiquitin–proteosome and lysosome–autophagy pathways [4,13]. The gut microbiota (also known as the gut microbiome) and its modulation seem to be the next frontier in PD treatment, as gut dysbiosis, which is an imbalance in the gut microbiota, is associated with the onset and progression of PD [14]. α-Synuclein aggregation, an inefficient lysosome–autophagy pathway, and Lewy body inclusions interplay and have prominent roles in PD progression [15,16]. It is now well understood that gut dysbiosis and pathogenesis in PD in humans are strongly associated, with several studies showing that individuals with PD exhibit a gut composition that is both different from that seen among healthy individuals and imbalanced in a manner promoting the pathologic components of PD [17,18]. Recent studies highlight the importance of flavonoids in bringing back balance in the gut microbiota, resetting GBA, alleviating neuroinflammation and neurodegeneration, and functional neuroprotection in preclinical models of CNS diseases, including PD. This review article focuses on recent research on three specific flavonoids, epigallocatechin-3-gallate (EGCG), quercetin, and kaempferol, known for most promising efficacy in the alleviation of gut dysbiosis and attenuation of multiple molecular mechanisms that cause pathogenesis in PD.

## 2. Functions of the Gut Microbiota in the Human Body

The human gut is a complex ecosystem consisting of nearly 100 trillion microbes such as bacteria, viruses, fungi, protozoa, archaeobacteria, and an array of other organisms [17]. Among these, bacteria tend to serve as the predominant part; however, the diversity that is exhibited among the bacterial phyla and species only goes on to add to the complexity of this ecosystem. The bacterial composition of the gut microbiota consists of the following seven phyla: *Firmicutes*, *Bacteroidetes*, *Actinobacteria*, *Fusobacteria*, *Proteobacteria*, *Verrucomicrobia*, and *Cyanobacteria*, with bacteria from the *Firmicutes* and *Bacteroidetes* phyla forming over 90% of the bacterial composition [19]. Together, these phyla contribute 300–500 different bacterial species to the gut microbiota, and this foundational degree of complexity is merely augmented by the variability that is seen from person to person [20]. The gut microbiota is not identical in all humans, with a great degree of variation being seen not only from person to person but also among individuals as they age and are impacted by different influential factors. During the gestational phase, prenatal stress, antibiotic treatment, and the duration of the pregnancy can impact the colonization of a newborn’s gut microbiota, while the mode of delivery dictates where the constituting microbes are acquired, with microbial acquisition occurring among the maternal gut in vaginal delivery and among the skin in cesareans [17,19]. After initial colonization, the gut microbiota continues to change and develop throughout infancy until an individual reaches around 2.5 years of age, a point at which the microbial composition resembles that of an adult, and relative stability is reached until later life [20]. Beyond this point, significant non-pathologic alterations in the gut microbiota tend to only be seen when there is a variation in environmental exposure, antibiotic use, or diet [17,19]. This semblance of stability that is achieved in a healthy adult gut is an essential characteristic of the microbiota because each bacterial component that comprises this ecosystem contributes to the overall function of the gut microbiota across varying systems in the human body (Table 1). The gut microbiota plays an extensive role within the body, performing metabolic, protective, and structural functions at both local and systemic levels through various pathways [19]. Due to the complexity of the gut microbiota and its regulatory power, it has been found to have great impacts on multiple systems within the body, and research into the gut microbiota has become more and more common in recent years, with a particular emphasis being placed on understanding the impact deviations from a stable microbial composition has on the body and the development of different diseases including PD.

### 2.1. Gut Dysbiosis and Its Implications in Different Disease States

Millenniums ago, Hippocrates first postulated that the gut served as the origin of all diseases, stating that ‘death sits in the bowels’, and from then on, further research into the topic went on to provide even more support to this belief [19,23]. The gut plays an expansive role in the body, modulating multiple systems throughout the human host, and because of this, pathology in the gut can proliferate beyond the gastrointestinal (GI) system and lead to an array of disease states [24]. Gut dysbiosis is one of the most discussed pathologic states in the gut in which the microbial composition of the microbiota is imbalanced. This deviation from a healthy and homeostatic composition tends to occur in one of three ways: (i) the relative number of beneficial bacteria has decreased, (ii) the relative number of pathologic bacteria has increased, or (iii) the overall composition of the gut microbiota has become less complex [13]. The gut is a fragile ecosystem, with proper function depending on the network of interactions taking place among the trillion of constituting microbes; therefore, imbalances like these dysregulate the whole ecosystem and disrupt the structures and pathways that are modulated by the gut, inducing changes within the body that lead to a disease state.

Among its many roles, the gut microbiota performs a protective function within the body, working through an array of mechanisms to control the risk of pathogenic invasion and promote proper immune function; thus, when the gut microbiota is in a state of imbalance, this vital layer of protection deteriorates, and the body can be plunged into several pathologic states. For example, tight junction complexes (zonula occludens, zonula adherens, and desmosomes) are regulated by both microbial species within the gut and the metabolites that are produced by them; therefore, an increase in the relative number of pathogenic bacteria capable of producing endotoxins or a decrease in the number of beneficial bacteria that produce barrier fortifying agents such as glutamine, tryptophan, and short-chain fatty acids (SCFAs) may result in a ‘leaky gut’ [19]. With a weakened endothelial layer, microbes from the gut can easily transverse the ‘barrier’, introducing inappropriate microorganisms into the circulatory system, and the implications of this pathogenic invasion can spread throughout the body. This exposure not only pushes the body towards a septic state but also goes on to compromise the blood–brain–barrier (BBB). Due to the presence of Toll-like receptors (TLRs) among its endothelial cells, the BBB is made susceptible to the endotoxic effects of lipopolysaccharides (LPS) from the Gram-negative bacteria of the gut and the pro-inflammatory cytokines that are activated by these bacteria [25]. As it is weakened, the BBB is more permeable, rendering the entire CNS susceptible to harmful substances within systemic circulation [25]. In addition to this, as the translocation of bacteria across the gut epithelium reaches an uncontrollable level, a leaky gut can create a state of constant inflammation within the body, and this inflammatory response is merely augmented as other protective mechanisms fail due to microbial imbalances in the gut [26]. The gut also plays an essential role when it comes to maturation of the innate immune system; thus, where gut dysbiosis can induce a pathologic immune response, it can also decrease the immune system’s ability to effectively clear infections, predisposing the host to opportunistic infections [26]. An imbalance in the gut microbiota increases the body’s propensity toward inflammation and infection, creating an environment within which various disease states can be produced and promoted. Because of this, gut dysbiosis has strongly been implicated in an array of CNS diseases, including PD.

Due to their mode of delivery, cesarean babies exhibit a gut microbial composition that is less complex, with colonization by *Bacteroidetes* spp., a predominate phylum among the gut microbiota, taking place later in life [19]. As early as infancy, deficiency in the composition of the gut microbiota dictates the development of different childhood diseases [19,24,27]. In a rodent study of autoimmune uveitis, it was found that animals positive for human leukocyte antigen B27 (HLA-B27), a specific cell surface protein commonly found on white blood cells and involved in immune system dysfunction, were highly susceptible to the acute form of this ocular disease and exhibited composition of the gut microbiota significantly different from the control group [24]. In another study focusing on pre-eclampsia (PE), also called toxemia, a condition in pregnant women characterized by proteinuria and edema, it was found that PE women experienced gestational hypertension and exhibited an inimitable gut microbiota composition when compared with healthy pregnant women [27]. Using both in situ hybridization and bioinformatic sort out, the bacterial gut compositions among experimental (67 PE patients) and control (85 normotensive females) groups were identified and compared, showing that the bacterial gut composition differed at both the phylum and genus levels among these two groups. In comparison to normotensive women, PE patients had greater amounts of harmful genera (*Clostridium*, *Dialister*, *Veillonella*, and *Fusobacterium*) in their gut microbiota, while the amounts of beneficial genera (*Lachnospira*, *Akkermansia*, and *Faecalibacterium*) were seemingly depleted, with many of the elevated genera proving to be associated with the manifestation of several clinical symptoms seen in PE. Alongside this clinical observational study, a fecal microbiota transplantation (FMT) experiment was conducted, further incriminating the involvement of imbalance in the gut microbiota in the development of PE [27]. FMT with fecal contents from PE donors into germ-free mice (GFM) throughout their gestational period showed the development of worsening hypertension and proteinuria, the common clinical symptoms of PE, whereas GFM treated with fecal donations from the normotensive group showed no such clinical symptoms. Throughout history, such modifications to the gut microbiota have proven to be an effective mechanism through which improvements in health can be attained. FMT procedures have been utilized in the treatment of different health conditions since the 4th century, and contemporary practices of using prebiotic supplementation have proven to be beneficial when it comes to attenuating memory and cognitive dysfunctions in different CNS diseases [19]. Understanding how wide an influence the gut has within the human body and what roles gut dysbiosis plays in different disease states, exploration of the abnormal gut microbiota has become a popular topic of emphasis in PD research in recent years, and this change in direction has led researchers to find that the gut and PD are connected through multiple pathways.

### 2.2. Changes in the Gut Microbiota in PD

The GBA is a bidirectional pathway that acts as a route of communication between the brain and the gut via endocrine, neural, immunological, and metabolic signaling [28]. The vagus nerve, which is the main nerve of the parasympathetic nervous system, is central within this network, with the dorsal motor nucleus of the vagus nerve (DMV) not only innervating the GI tract but also allowing for axonal transport among its vagal projections due to its unmyelinated nature. This creates the means through which the gut and its surrounding enteric nervous system can communicate with the CNS [4,14]. As research into the GBA has rapidly progressed, its efficacy as a route of communication between these two biological systems has not only been validated but also implicated gut dysbiosis in the development and pathogenesis of PD. Studies suggest that the GBA, due to this anatomical connection, allows neurotransmitters and metabolites produced within the gut to circumvent the BBB, making it so the gut microbiota has the capacity to modulate brain function and physiology [28]. In addition to this, it has been suggested that the DMV potentially degenerates before the midbrain SN does. This indicates that the pathologic factors involved in the development of PD possibly utilize the GBA to spread to the brain, impacting sites among this pathway during the early stages of this disease [4]. This thought process is further supported by studies showing α-synuclein aggregates spreading in a defined manner from the enteric nervous system and olfactory bulb to the CNS [17]. Such findings have been foundational among emerging theories for implicating the gut microbiota in the development and pathogenesis of PD, and further recent research into the topic is providing additional support.

Following the detection of gut dysbiosis among individuals with AD, gut composition analysis has shown an overall pro-inflammatory profile among such neurodegenerative conditions [28]. A study in an animal model of PD clearly showed that the gut microbiota was responsible for regulating motor deficits and neuroinflammation [29]. Gut dysbiosis is a common clinical presentation in most neurodegenerative conditions, and research into PD specifically has shown that alteration in the composition of the gut microbiota is a unique feature in its presentation as well. Individuals with PD exhibit an increase in pro-inflammatory bacteria in their gut microbiota, while levels of beneficial bacteria such as *Prevotellaceae* fall, resulting in reduced mucin production in the gut. In addition to this, mutations in the SCNA (synuclein-alpha) gene seen in cases of early onset PD has been associated with an increase in opportunistic pathogens in the gut microbiota of these patients. Such changes in gut composition potentially contribute to the disease state, with diminishing mucin levels weakening one of the gut’s primary layers of fortification and the subsequent escape of pathogenic microbes as well as pro-inflammatory factors into the systemic circulation promoting the chronic inflammation seen in PD [17,30]. A recent analysis has even shown that nearly half of underweight PD cases are the result of changes in the gut microbiota [17]. Recently, the development of therapeutics based on the gut microbiota is gaining popularity in the treatment of PD [31]. Gut dysbiosis is currently seen as a driving force in the pathogenesis of PD rather than a symptom of the disease, and this belief has been further supported as FMT studies utilizing samples from PD donors and mice models of PD alike have been conducted. In one such study, mice were administered fecal pellets from either a vehicle group of mice or a group of mice with rotenone-induced PD [32]. Findings showed that the mice administered with fecal pellets from the rotenone-induced PD group exhibited gut dysbiosis characterized by a decrease in bacterial diversity, microglial activation and inflammation, and degeneration of dopaminergic neurons in the SN, while the mice administered with fecal pellets from the vehicle group did not exhibit any of these rotenone-induced PD symptoms. In another FMT study, it was found that fecal transplants from PD individuals enhanced motor dysfunctions in an ASO (alpha-synuclein overexpressing) mice model, as indicated by four different motor function tests (i.e., beam transversal, pole descent, nasal adhesive removal, and hindlimb clasping reflex) and resulted in the development of several pathologic features commonly seen in PD [29]. In comparison to germ-free ASO mice, the ASO mice that underwent fecal transplantation exhibited GI impairment, constipation, and drastically higher levels of alpha-synuclein aggregates among the caudoputamen and substantia nigra, as assessed by immunofluorescence spectroscopy. Levels of the α-synuclein protein and transcript were compared among the two groups to verify that the variation seen in spectroscopy findings was not the result of differences in transgene expression rather than treatment. Treatment with antibiotics went on to further validate the causal effects of FMT in the experimental group, for the pathologic state induced was seemingly reversed, leaving the experimental group to strongly resemble their germ-free counterparts [29]. Gut dysbiosis has seemingly been implicated in the development of PD, time and time again, leading researchers to try to better understand the mechanisms through which pathogenesis in PD occurs.

Although the exact etiology behind PD is still mysterious, growing knowledge on the topic has led researchers to find that PD is most likely a multifactorial disease whose progression, as well as pathological presentation, is the result of chronic inflammation, oxidative stress, and dysfunction in the ubiquitin–peroxisome and lysosome–autophagy pathways. Together, these pathologic factors culminate into a progressive neurodegenerative state, resulting in the DA deficiency and subsequent motor dysfunction seen in this disease. Understanding the involvement of alterations in the gut microbiota in the development and promotion of such etiologic factors in PD is an area of intense focus because such associations can serve as an avenue in devising potential therapeutic modalities for adjustment of the gut microbiota and thereby blocking the process of development and progression of PD. Through research in this manner, it has already been found that more than 50% of the body’s DA originates from the gut; thus, disruption to the gut and its ability to produce neurotransmitters may sufficiently contribute to deficiencies in DA in PD cases [19]. In addition to this, the gut, in a state of dysbiosis, seemingly promotes the aggregation of the α-synuclein protein, with the formation of such aggregates appearing among organs of the GI system and the enteric nervous system [17]. Thus, imbalances in the gut microbiota contribute to the accumulation of toxic aggregates within the body, subsequently causing inflammatory effects that are induced in areas in which they are present. The pathologic presentation of gut dysbiosis goes on to further promote inflammation throughout the body, as pro-inflammatory components such as LPS and cell capsule carbohydrates released from gut bacteria and the by-products produced by these organisms circumvent the weakened gut barrier seen in states of dysbiosis and make their way into circulation where an intense inflammatory response is mounted [13].

Overall, an imbalance in the gut microbiota has been proven to be highly implicated in PD development, with gut dysbiosis serving as a common clinical sign among individuals with PD and exasperating the etiologic factors associated with this condition. Therefore, developing or discovering any novel treatment that can restore a healthy and homeostatic state in the gut while directly attenuating the pathologic factors that drive the neurodegenerative process in PD would serve as a promising frontier when it comes to treating this condition in a manner that goes beyond the management of symptoms.

## 3. Flavonoids, Classification, and Structures

Flavonoids (also called bioflavonoids) are naturally occurring organic compounds that constitute the largest subclass within the polyphenol family, contributing to approximately 60% of all polyphenols found in nature [33]. This polyphenol class is further subdivided into six major classes: flavan-3-ols, flavonols, flavanones, flavones, anthocyanins, and isoflavones [33]. The subdivision of flavonoids into these various subclasses is dictated by structure, with each subclass being grouped based on shared similarities among ring connections, ring structures, and ring substitution patterns. At its base structure, all flavonoids consist of a basic carbon skeleton of C6-C3-C6, with three carbon atoms in an oxygenated heterocyclic conformation binding together two aromatic rings [30,34,35]. As the secondary metabolites of plants, flavonoids enrich a variety of fruits and vegetables, with high concentrations of various flavonoids being seen among citrus fruits and other colorful fruits, medicinal plants, herbs, legumes, and many more plant types [36]. Flavonoids play an important role in the plant kingdom, with their synthesis occurring when plants are placed in adverse situations or physiologically stressful environments to act as a layer of defense against stressors (e.g., frost, drought, heat acclimation, UV radiation, etc.) [37,38]. This class of organic compounds behave as potent defensive agents under various conditions, and this effect has proven to be quite reproducible in humans upon consumption of flavonoid-enriched vegetables, fruits, and herbs. In many countries across the world, the consumption of flavonoids as supplements or flavonoid-rich diets has been implemented as a common health practice, with Asian and Mediterranean populations exhibiting some of the greatest rates of consumption due to their flavonoid-enriched fruit and vegetable diets, especially in comparison to populations in the Western world [28,33]. Population studies have even shown that Asian and Mediterranean diets prevent a variety of pathologies associated with aging and age-related diseases, especially diseases of the CNS [30]. For millennia, plants such as garlic, turmeric, green tea, and saffron have been consumed and utilized for their potential health benefits, and it is now understood that polyphenols contribute to their perceived health benefits and therapeutic effects [39]. Flavonoids exhibit an array of proposed health benefits, with these polyphenolic compounds behaving as natural gut modulators, antioxidants, free-radical scavengers, anti-inflammatory agents, neuroprotective molecules, and much more (Table 2). Because of this, some major flavonoid subclasses have quickly become intriguing targets when it comes to the exploration of new potential therapeutic agents for the treatment of PD, especially focusing on the amelioration of gut dysbiosis in this major CNS disease [28,33].

### 3.1. Flavonoids and the Gut Microbiota Work in a Symbiotic Relationship

Understanding how extensively gut dysbiosis is implicated in the development and progression of PD and exploring the ability of flavonoids to positively modulate the gut microbiota is fundamental in establishing the use of this polyphenolic class as a new frontier in the treatment of PD in preclinical models and clinical settings. Among their many proposed health benefits, flavonoids have been shown to promote a homeostatic state within the human gut. As stated before, gut dysbiosis is characterized by a decrease in the relative number of beneficial bacteria, an increase in the relative number of pathologic bacteria, and an overall loss of complexity among microbial composition, and it is through direct reversal of such pathologic changes that flavonoids have been found to attenuate gut dysbiosis [13]. Flavonoids behave like natural prebiotics, promoting the growth of healthy and beneficial bacteria in the gut [28]. Catechin flavonoids from green tea have been shown to increase the relative number of *Akkermansia muciniphila* in the composition of gut microbiota, resulting in enhanced gut integrity and reduced inflammation as observed in the mouse models, while flavonoid modulation in the gut, in general, has been found to improve gut barrier function and reduce inflammation through increases in mucus and SCFAs such as acetate, propionate, and butyrate produced by gut bacteria [28,33]. In addition to the promotion of beneficial bacteria and production of SCFAs, flavonoids like anthocyanins have been shown to stimulate the elimination of pathologic strains of bacteria (e.g., *Staphylococcus aureus* and *Salmonella typhimurium*) within the colon [28]. However, this relationship is not commensal for flavonoid–gut interactions, which have also been shown to have some potential benefits when it comes to promoting the bioactivity of flavonoids within the body. It has been proposed that several of the neuroprotective benefits associated with nutrient consumption are at least in part dependent upon the generation of bioactive metabolites by the gut microbiota, and this is a concept that seemingly holds true when one looks at the bioactivity of flavonoids [33]. Polyphenols are often inactive in their dietary form and must undergo metabolic changes by the gut microbiota (e.g., removal of sugar moieties) to display their active form [21]. For example, quercetin is commonly present as glycosides (e.g., rutin) among dietary sources and must undergo deglycosylation in the human small intestine mucosa (using lactase-phlorizin hydrolase localized to the apical membrane of small intestinal epithelial cells) or colon (using β-glucosidase derived from the gut microbiota) to be absorbed [40]. Flavonoids are known to undergo extensive metabolization within the GI system, undergoing initial metabolization within the intestine and further processing within the liver, which plays a large part in determining the bioavailability of flavonoids and contributes to the biological efficacy of these polyphenols [34]. Despite their low bioavailability, flavonoids have shown positive biological outcomes in several studies, leading researchers to believe that metabolites produced through biotransformation may prove to be biologically active as well and induce similar physiological effects as their parent compound, a phenomenon that is today referred to as the low bioavailability/high bioactivity paradox [40]. For instance, metabolites produced in the gut-induced modification of anthocyanins exhibit high antioxidant capacity in the peripheral and local (i.e., gut) systems. In other situations, it has even been found that such flavonoid-derived metabolites can outcompete their parent compound in terms of bioactivity, eliciting more potent effects within the body [28,40]. This strong symbiotic relationship that exists between flavonoids and the gut microbiota offers support for the future use of flavonoids in the treatment of PD, for it not only illustrates an indirect pathway through which flavonoid administration can attenuate both the pathogenesis and progression of PD through the gut but also provides a means through which the direct effects of flavonoids among the body are potentially promoted.

### 3.2. Potential of Flavonoids in the Treatment of PD

**Figure 3:**
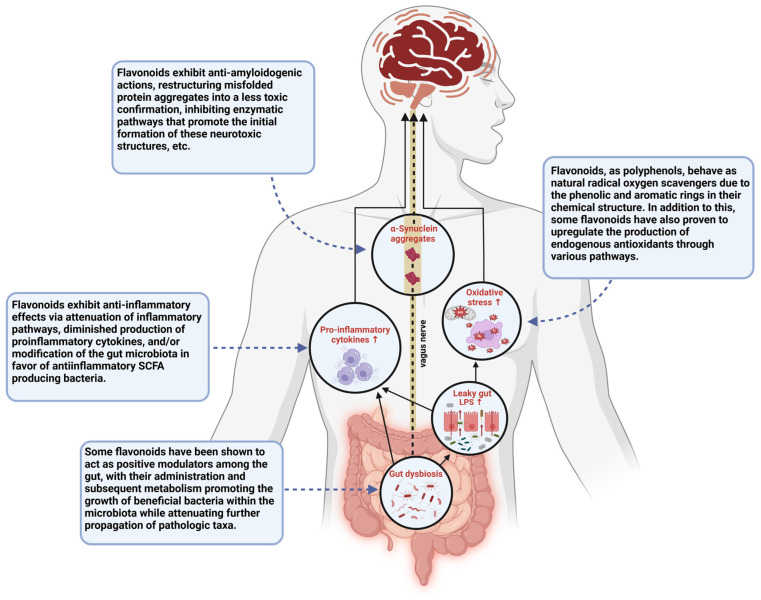
This schematic diagram illustrates various pathways through which gut dysbiosis contributes to the disease state seen in PD and the points at which flavonoid administration has been suggested to show therapeutic potential in the treatment of PD. This figure was created using BioRender.

The biological efficacy of flavonoids has been explored in relation to an array of neurodegenerative and inflammatory conditions. Studies have shown that sustaining a flavonoid-rich diet may be preventative when it comes to developing inflammatory diseases, and the neuroprotective capacity of these polyphenolic compounds has gone on to garner support among researchers as well [33,34]. Through a cross-sectional study of stroke-free subjects from the Framingham Heart Study Offspring Cohort (mean age: 60.6 years), it was found that the total daily amount of flavonoid consumption among participants was inversely related to neurodegeneration, as measured by the white matter hyper-intensities volume in their magnetic resonance imaging (MRI) scans [33,41]. This serves as an indication that regular consumption of flavonoids may produce some visible neuroprotective effects that deter progressive neurodegeneration over time. In relation to PD specifically, flavonoid consumption has been shown to be associated with a lower risk of developing PD among men, and though findings among women have not yet proven to be statistically significant, studies in which the sexes were pooled suggested that overall, high consumption of berries, which are widely known flavonoid-rich fruits, reduces the risk of PD [30,42]. In addition to their potential preventative capabilities, flavonoids possess an array of characteristics that enable them to directly attenuate the key pathogenic mechanisms involved in the initiation and expansion of PD (Figure 3) [17,28,30,33]. Flavonoids behave as natural, free-radical scavengers, with phenolic hydrogens among their structure, rendering them good hydrogen-donating molecules. In addition to this, these are known to be anti-inflammatory agents, capable of attenuating pro-inflammatory pathways by inhibiting regulatory enzymes and transcription factors involved in the activation and progression of inflammatory cascades [34]. Flavonoids combat dopamine reduction, reduce the formation of reactive oxygen species, attenuate mitochondrial dysfunction, and exhibit an array of other biological activities related to the diminishing pathogenesis of PD [30,38]. As more research into these polyphenolic compounds is being conducted, their administration is clearly showing a strong prospective therapeutic approach for the treatment of PD, and an emphasis is seemingly being placed on certain flavonoids due to their perceived efficacy in this regard. Amidst these flavonoids, the most promising are EGCG, quercetin, and kaempferol.

Among the flavan-3-ol subclass is EGCG, a catechin predominately found in green tea [33]. As a flavonoid, it has been proposed that EGCG stimulates anti-inflammatory, antioxidant, and neuroprotective effects; however, studies have shown that the therapeutic potential of this flavonoid extends beyond these fundamental characteristics. In addition to such beneficial aspects commonly associated with flavonoids, EGCG has been shown to exert positive effects in pathologic mechanisms that are specific to neurodegenerative conditions like PD. For example, EGCG exhibits specific anti-amyloidogenic actions that, within the cases of PD, may be able to ameliorate the pathologic accumulation of α-synuclein aggregates in Lewy body inclusions [28]. The potential anti-amyloidogenic effects of this flavonoid are further augmented by its structure, for the 3,4,5-trihydroxy substitution pattern seen among flavonoids like EGCG has been proposed to produce proteosome activity, as evidenced in some in vitro studies [13]. Studies have also shown that prophylactic administration of EGCG in rat models of PD rescues the animals from developing PD symptoms upon injection with rotenone, a chemical commonly used in the creation of animal models of PD [28].

The second flavonoid that is focused on within this review article is quercetin, the most common flavonol in nature [28]. Quercetin has been shown to behave as a potent neuroprotective agent amid several studies of neurodegenerative disease. In different animal models of AD, it was found that quercetin was efficacious when it came to ameliorating several of pathologic factors commonly seen among not only AD but also PD [28,42]. In addition to this, administration of quercetin has been shown to protect against the pathologic effects of 1-methyl-4-phenylpyridinium (MPP^+^)-induced mitochondrial dysfunction and LPS-induced inflammation among in vitro studies and reduce ROS levels, further demonstrating the potential therapeutic role this flavonoid may have in PD. Quercetin has even been shown to promote mitophagy, and this distinctive feature potentially attributes, at least in part, to the positive effects produced in such studies, for controlled elimination of the damaged or dysfunctional mitochondria can alleviate their pathological effects within the body and among neighboring healthy organelles [28,40]. During periods of oxidative stress, quercetin has also been shown to maintain levels of glutathione, one of the body’s most potent endogenous antioxidants, creating a means through which its own fundamental antioxidant capabilities are expanded [30]. This flavonoid can also circumvent the BBB; thus, its beneficial effects can be exhibited in the brain, the primary site of pathogenicity in the case of PD [43].

Kaempferol serves as the third and final flavonoid of focus in this article. Through the regulation of oxidative stress, neuroinflammation, apoptosis, and α-synuclein aggregation, this flavonol and its derivatives have been suggested to produce an array of neuroprotective effects in PD [44]. In a study involving a transgenic *Drosophila* model that mimics the pathologic signs of PD (e.g., α-synuclein aggregation, dopaminergic degeneration, and motor dysfunction), the administration of kaempferol at various doses was found to attenuate several of the etiologic factors related to PD in a dose-dependent manner [45]. Markers of oxidative stress declined in the treatment group alongside the activity of endogenous antioxidant enzymes (e.g., glutathione S transferase, superoxide dismutase, and catalase), indicating that the ROS load attributed to PD was diminished significantly in the face of kaempferol administration. In addition to this, both the activity of caspase-9 (a cysteine protease for induction of apoptosis) and neuronal apoptosis in the brain declined in a dose-dependent manner, suggesting that kaempferol administration attenuated the occurrence of apoptosis due to its neuroprotective effects. This was further supported by the fact that levels of tyrosine hydroxylase, the rate-limiting enzyme involved in dopamine production from L-Dopa in the nigrostriatal pathway, were increased in the experimental group. In addition to this, kaempferol, in a dose-dependent manner, delayed motor dysfunction and an array of other PD symptoms. Much like quercetin, kaempferol can induce its effects amidst the brain due to the capability of its BBB permeability [45]. Recent attempts at developing novel drugs for PD treatment have focused on directly targeting the disease-causing factors rather than compensating for its pathologic outcomes through dopamine supplementation [46].

Through the exploration of flavonoids like EGCG, quercetin, and kaempferol and the mechanisms through which they work within the body, it seems as if, right within nature, researchers today may be able to discover an impactful therapeutic treatment for PD.

## 4. Neuroinflammation in PD

Under normal conditions, inflammation plays a protective role throughout the body; however, when inflammatory pathways go unrestrained and are left in a state of constitutive activation, the overall effects become deleterious [46,47]. Then, neuroinflammation changes from a neuroprotective mechanism employed amongst our CNS to a destructive one, and this pathologic phenomenon is a marked characteristic seen throughout the development and progression of PD. Amid this condition, constitutively activated microglia in the brain secrete pro-inflammatory cytokines that promote inflammatory deregulation, leading to systemic damage, and this pathologic effect is merely perpetuated as ROS and other free radicals produced during the inflammatory response induce further damage [34,48]. A self-sustaining vicious cycle of inflammation develops, and this largely contributes to the neurodegeneration seen throughout the progression of PD. A major player in the initiation of this inflammatory cascade is the nucleotide-binding domain, leucine-rich repeat (NLR) family pyrin domain containing 3 (in short, NLRP3) macromolecule, a well-understood inflammasome that persuades the maturation and secretion of pro-inflammatory cytokines such as interleukin-1β (IL-1β) and IL-18, with aberrant activation of this inflammasome complex proving to be implicated in the development of neurodegenerative and age-related CNS diseases like PD [42,43,49]. Inflammasomes are the pattern recognition enzymes among the innate immune system for eliciting activation of inflammatory caspases and processing of IL-1β. Activation of the NLPR3 receptor in immune and epithelial cells occurs via recognition of endogenous danger-associated molecular patterns (DAMPs, such as uric acid crystals, ATP, β-amyloid plaques, and other indicators of physiologic damage) and exogenous pathogen-associated molecular patterns (PAMPs, such as protozoan, viruses, fungi, etc.) factors; thus, cellular degeneration, which occurs in the hub of PD, and gut dysbiosis-induced bacterial products released into the circulatory system potentially feed into this pathway, further promoting the inflammation seen in this disease [49]. Neuroinflammation is a strong driving force in the progressive pathogenesis of PD; therefore, the attenuation of this pathologic component may serve as an intriguing target among potential PD treatment pathways.

### Gut Dysbiosis in Promoting Neuroinflammation in PD

Dysbiosis in the gut weakens both the gut membrane and BBB and thus creates a pathway through which bacterial components and the metabolites they produce can enter systemic circulation. Plasma levels of LPS, a highly cytotoxic component of Gram-negative bacteria, have been found to be elevated among individuals with PD due to gut dysbiosis-induced barrier dysfunction, and this results in a robust inflammatory immune response that contributes to the neurodegeneration seen in PD [17]. Exposure to endotoxins like LPS elevates plasma levels of pro-inflammatory mediators, promotes immune cell adhesion and migration, and activates microglia through the binding of TLR4, inducing a state of systemic inflammatory dysfunction within the body [17,50,51]. Looking at the effects of a ‘leaky gut’ among individuals with this condition, the assumption that the gut is involved in the development of neuroinflammation has garnered some support, and more researchers recently have only added to this postulation as additional facts have been gathered about SCFAs and their role in this.

**Figure 4:**
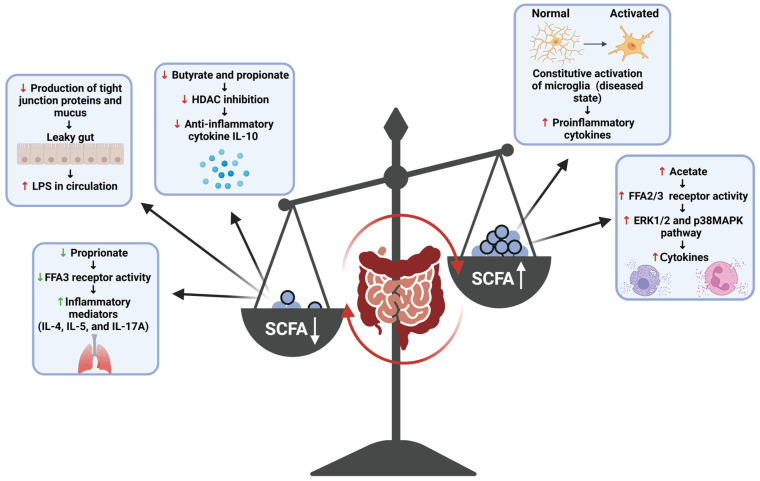
This schematic diagram highlights the pleiotropic effects of SCFAs and the inflammatory state that can be induced due to overproduction or underproduction of SCFAs in an unbalanced human gut. FFAs = free fatty acids; ERK = extracellular-signal-regulated kinase; MAPK = mitogen-activated protein kinase; IL = interleukin. This figure was created using BioRender.

SCFAs are small compounds produced through the gut bacteria-dependent breakdown of consumed dietary carbohydrates and proteins [17]. SCFAs can modify the activity of immune and endothelial cells through receptor binding and histone modification [51]. Acetic, propionic, and butyric acid (including their salt forms: acetate, propionate, and butyrate) serve as the predominate forms of SCFAs produced by the gut microbiota, and thus their roles within the body are most appreciated and comprehended by the scientific community [17]. Research into these three SCFA compounds has shown that much like the inflammatory responses they mediate, these compounds play a dichotomous (opposing or contradictory) role within the body, with their interaction with receptors and enzymes either promoting or dampening various inflammatory pathways (Figure 4) [48,51,52]. Generally, butyrate is known to attenuate inflammatory pathways and inflammation-promoting states through various mechanisms. Within the gut, butyrate promotes the production of tight junction proteins and mucus, supporting the structural integrity of the epithelial membrane; thus, the production of this metabolite serves as a means through which the inflammatory effects of a ‘leaky gut’ can be diminished [52]. In addition to this, studies have shown that interactions of butyrate with immune cell receptors and histone deacetylase (HDAC) enzymes have also been shown to induce anti-inflammatory effects. The binding of this SCFA to the free fatty acid receptor 3 (FFA3) and GPR109A (a G protein-coupled receptor for nicotinate, but GPR109A recognizes butyrate with low affinity) of immune cells induces anti-inflammatory effects in macrophages and the colon, respectively, while the inhibition of HDAC by butyrate has been shown to result in anti-inflammatory effects among macrophages and dendritic cells [51]. Β-Hydroxybutyrate, a ketone metabolite, has also been shown to attenuate production of the pro-inflammatory cytokines IL-1β and IL-18 by NLPR3 activation without eliciting any off-target immunosuppressive effects and thus provides a means through which the effects of this inflammasome complex can be dampened among cases of PD [49]. Butyrate is an important anti-inflammatory agent; thus, reduced levels of SCFA-producing bacteria increase the pro-inflammatory response in PD. It is a decrease in butyrate production that is mostly concerned with the development of gut-induced PD symptoms [17]. SCFAs have also been shown to behave as pro-inflammatory compounds in the body. Some studies have shown that acetate activates the FFA2 and FFA3 receptors and induces cytokine production via the activation of other pro-inflammatory pathways (e.g., ERK1/2 and p38 MAPK) and butyrate can augment the activation of the nuclear factor kappa-light-chain-enhancer of activated B cells (NF-κB), a potent pro-inflammatory mediator, by increasing the expression of specific TLR among human Langerhans cells [51,53]. SCFAs are pleiotropic mediators of inflammation; however, their production at normal ratios in the body has proven to be beneficial overall when it comes to attenuating inflammation; thus, the promotion of their production by commensal gut bacteria serves as one of the plausible mechanisms through which neuroinflammation in the case of PD can be attenuated through the gut.

## 5. Mitochondrial Dysfunction and Oxidative Stress in PD

In 1982, William Langston assessed seven patients in the San Francisco Bay area after they began to exhibit Parkinsonian-like symptoms following abuse of synthetic heroin [4]. Studies of the case eventually found that 1-methyl-4-phenyl-1,2,3,6-tetrahydropyridine (MPTP), a byproduct seen in unregulated opioid production, was responsible for the rapid development of Parkinsonian symptoms in those seven individuals, strongly implicating mitochondrial dysfunction in the development of PD [5]. In the brain, MPTP is converted into MPP^+^, a potent complex I inhibitor, which can enter the dopaminergic neurons via DA transporters. Inhibition of complex I in the mitochondrial electron transport chain initiates a pathologic cascade in which electron leakage leads to rising ROS levels, and as a result, microglial cells are constitutively activated, causing neurodegeneration in the nigrostriatal pathway that is identical to what is seen in PD [30,54]. The effects of this complex I inhibitor have proven to be so like the pathologic state seen in PD that to this day, MPTP is the only neurotoxic agent known to cause a clinical presentation that is indistinguishable from PD, down to the development of L-Dopa responsive motor dysfunction [1]. Exposure to complexes I inhibitors such as MPTP and rotenone is now commonly used in the creation of animal models of PD due to the Parkinsonian-like effects these chemicals induce, illustrating how significant a role mitochondrial dysfunction plays in the Parkinsonian etiology [5,55]. Additional evidence implicating mitochondrial dysfunction in the development and progression of PD can be found by merely looking among the individuals who suffer from this condition. Studies have shown that PD patients exhibit reduced mitochondrial complex I activity, with mitochondrial dysfunction being seen in the peripheral monocytes of these individuals early in the development of the disease [4,46]. In addition to this, many of the genes that are associated with monogenic cases of PD (e.g., PARK 2/Parkin, PINK-1, DJ-1, and LRRKS) encode for proteins that regulate mitochondrial function and ROS homeostasis and, when mutated, induce symptoms that bare a strong resemblance to the motor/postural abnormalities seen in PD (e.g., dystonic gait, leg tremor, L-Dopa responsive dystonia, freezing, etc.) [4,50]. In a postmortem study of PD patients, it was even shown that levels of the nuclear factor erythroid 2-related factor 1 (Nrf1), also known as nuclear factor erythroid-2-like 1 (NFE2L1), a protein involved in the regulation of mitochondrial function, were drastically reduced or even absent [56]. Mitochondrial dysfunction has proven to be an important etiological factor in PD; however, this relationship does not only implicate this one pathological component alone, for it is known that the electron transport chain is the primary producer of ROS in the body and, thus, with mitochondrial dysfunction comes oxidative stress [30].

Studies have shown that oxidative stress levels are elevated in the SN of PD patients, where mitochondrial dysfunction plays a large role; furthermore, many other pathologic states seen in PD have been found to feed into this etiologic component [47]. Iron is present in excess in the SN of PD patients, with the ratio of reduced to oxidized iron reaching 1:3 (the normal ratio is 1:1) in this region of the brain [30,47]. As iron begins to accumulate, hydroxyl radicals (•OH, the highly reactive free radicals) start to form as the metal readily reacts with dopamine and hydrogen peroxide (Fenton reaction) present in the SN, resulting in a high level of oxidative stress [55]. Due to their highly reactive nature, free radicals alter the structural conformation of lipids and other functional macromolecules, damaging them via cross-linking and polymerization [34,35]. High plasma levels of lipid peroxidation biomarkers are found in PD individuals, and ROS-mediated damage is seen in PD postmortem studies [30,57]. This issue is most likely further exasperated by the diminishing levels of endogenous antioxidants, for studies have shown that PD severity and the level of glutathione (GSH) loss are positively correlated and that decreasing GSH levels often precede other PD-associated changes in the SN [50]. Therapeutic targeting of mitochondrial dysfunctions and ROS is currently considered an important strategy for the treatment of neurodegenerative diseases [58]. As mitochondrial dysfunction and oxidative stress play impactful roles in the development of PD, several clinical trials in recent years have focused on these etiologic factors (Table 3) for the development of new treatments for PD.

### Gut Dysbiosis in Mitochondrial Dysfunction and Oxidative Stress in PD

Much like in the case of neuroinflammation, the mortification of membrane integrity seen in the gut and BBB during gut dysbiosis augments mitochondrial dysfunction and oxidative stress in PD. With a leaky BBB, iron accumulation can worsen in the substantia nigra of PD patients, resulting in mounting levels of oxidative stress and worsening mitochondrial dysfunction as the iron overload begins to disrupt the electron transport chain and produce free radicals [30,35]. In addition to this, the immune response that is induced upon exposure to bacterial endotoxins in the circulatory system not only results in inflammation but also feeds into the oxidative stress issue as ROS are released from macrophages and other granulocytic lymphocytes. Specifically, the presence of LPS has been implicated in this process the most, for studies have shown that through the stimulated production of inducible nitric oxide synthase (iNOS), superoxide, and peroxynitrite, this endotoxin augments levels of ceramide within the body, a lipid molecule that has been shown to negatively impact mitochondrial function [59,60]. However, amidst the gut microbiota, the production of butyrate has been shown to serve as a potential mechanism through which mitochondrial dysfunction and oxidative stress can be attenuated, for studies have shown that this SCFA diminishes the impact ceramide has on mitochondrial function [57]. By converting ceramide into a protective ganglioside, butyrate lowers levels of this lipid molecule within the body, working against the ROS and iNOS stimulation of ceramide production that is seen during periods of high oxidative stress.

## 6. Toxic α-Synuclein Aggregation and Autophagy Impairment in PD

The formation of α-synuclein aggregates amidst Lewy body inclusions is a molecular hallmark in PD, with the presentation of this clinical symptom proving to be of such importance that to this day, the presence of Lewy bodies is required for postmortem confirmation of PD diagnosis [50]. The pathologic effects induced by this protein stem from its non-amyloid beta (NAC) domain, for this hydrophobic region is amyloidogenic and thus predisposes the α-synuclein protein to misfold and aggregate in its wild-type and mutant forms [30,50,61]. Aggregation of this protein takes one of three distinct forms: (i) oligomers, (ii) protofibrils, and (iii) fibrils; however, it is the oligomeric form that induces neurotoxic effects in dopaminergic neurons [30,46]. Excess uptake of α-synuclein oligomers by astrocytes in the CNS causes pro-inflammatory factors to increase in number and mitochondrial dysfunction to worsen as the lysosomal degradation pathway begins to break down in PD [46]. Partially degraded, these α-synuclein structures coalesce into the Lewy body formations as seen in PD, and their pathological effects within the brain persist [4,30]. This protein aggregate has been implicated in the development of every known pathologic presentation seen in PD, having been linked to lysosomal and mitochondrial dysfunction, autophagy impairments, excessive inflammatory responses, nigral degeneration, and the development of L-Dopa-responsive motor abnormalities [4]. Even the preventative effects of nicotine have been shown to emphasize the importance of this etiologic factor, for it has been suggested that the potential preventative mechanism through which it works is anti-amyloidogenic in nature [39]. α-synuclein aggregation is a key etiological factor in PD; however, its importance does not dampen that of autophagy impairment, for the two go hand-in-hand. As previously mentioned, dysfunction of the lysosome–autophagy pathway is a key driving force in the formation and persistence of the Lewy body inclusions in the brain, and the importance of this pathologic component only garners further support as one investigates PD-associated diseases. Gaucher’s disease, a lysosomal storage disorder in which the breakdown of glucocerebroside is mitigated, has been shown to significantly increase an individual’s propensity to develop PD [4]. In addition to this, a mutation in the Parkin gene, a common cause of monogenic PD, has been implicated in the disruption of the ubiquitin–proteosome pathway, indicating that such impairments in the autophagy and proteosome pathways play a prominent role in the development and progression of this condition [17]. Both the aggregation of α-synuclein proteins and autophagy impairment have been shown to act as important etiologic components in progressive pathogenesis in PD; thus, the direct attenuation of these pathologic factors would serve as an interesting mechanism of action among potential PD therapeutic approaches.

### The Gut Microbiota in α-Synuclein Aggregation and Autophagy Impairment in PD

A recent study demonstrated that a gut bacterial metabolic pathway is responsible for α-synuclein aggregation and pathogenesis in PD [62]. It has been suggested that the propagation of α-synuclein aggregates in the body occurs in a prion-like manner, with the initial development of these neurotoxic structures occurring in the gut and their subsequent spread to the brain occurring via the dorsal motor nucleus of the vagus nerve [17]. This theory was first proposed in a paper by Braak et al., in which the spread of Lewy bodies throughout PD development was described as a predictable pattern of propagation [14]. It has been stated that after the invasion of some unknown pathogen permeable to the GI membrane occurs, aggregates spread through the enteric nervous system (ENS), up the afferent projections of the vagus nerve, and into the dorsal motor nucleus, where they continue to propagate throughout the brain until the point is reached where Lewy bodies can be seen amongst the pars compacta of the substantia nigra and cerebral cortex in the final stages of the PD. The dorsal motor nucleus was found to serve as the ideal route of transport among this theorized pathway, for the GBA has shown that the vagus nerve’s composition of long and thin unmyelinated axons makes it so retrograde axonal transport among this nerve is easy not only for hormones and other neuroactive substances but for pathogens as well [14]. With this single theory, the gut was implicated in the development and propagation of α-synuclein aggregates, providing researchers with a better understanding of the pathway this pathological factor takes throughout the progression of this disease and a potential point of origin that can be targeted into the administration of flavonoids as the future of anti-amyloidogenic treatments.

## 7. Anti-Inflammatory Effects of ECCG, Quercetin, and Kaempferol

At this point, it is well understood that neuroinflammation is a key pathologic component of PD thus understanding the anti-inflammatory capabilities of some specific flavonoids, EGCG, quercetin, and kaempferol is fundamental when it comes to their potential application as PD therapeutics. Even outside the realm of PD, the anti-inflammatory properties of these polyphenolic compounds serve as the basis of their beneficial effects alongside their antioxidant abilities [28]. Flavonoids are known to behave as potent anti-inflammatory agents, and through several studies, it has been shown that these effects are not induced through one sole mechanism of action [33]. These compounds have been found to reduce the expression of the enzymes involved in arachidonic acid metabolism, inducing NSAID (nonsteroidal anti-inflammatory drugs) like effects in the body; some studies on NSAID use have been proposed to potentially induce preventative and neuroprotective effects in some cases of PD, though further research is needed [4,34,47]. Quercetin has been found to attenuate the production of inflammatory mediators among LPS-activated BV-2 microglia, while kaempferol has been shown to dampen inflammatory pathways by suppressing the expression of pro-inflammatory transcription factors and adhesion molecules [42,63]. A recent study suggests that kaempferol provides its anti-inflammatory effects by accelerating the development of T regulatory (Treg) cells [64]. Flavonoids induce their anti-inflammatory properties through an array of mechanisms; thus, by better understanding the pathways through which they work, the implementation of quercetin, EGCG, and kaempferol in inflammatory conditions like PD can be better supported.

### NF-κB Pathway Targeting by EGCG, Quercetin, and Kaempferol

**Figure 5:**
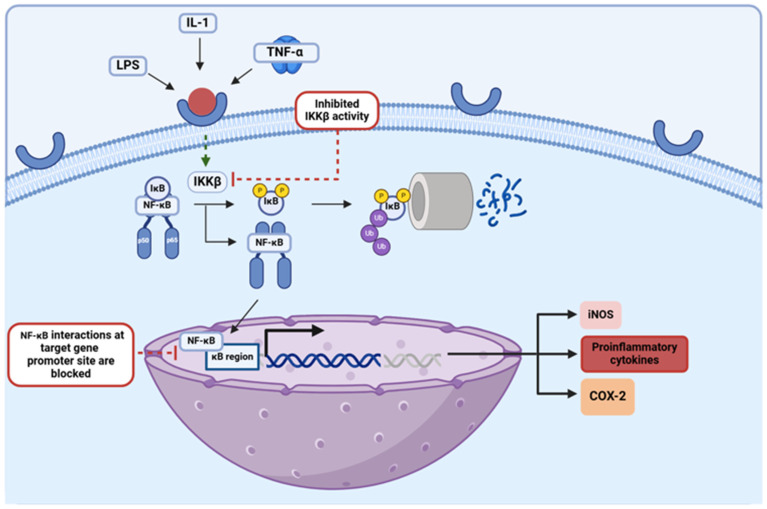
Schematic depiction of the NF-κB pathway and two points at which flavonoids have been shown to directly inhibit it. This figure was created using BioRender.

The NF-κB transcription factor regulates the expression of multiple pro-inflammatory genes, serving as the central hub of various inflammatory pathways in the body [51]. In the absence of the inhibitor of the kappa B (IκB) protein, an inhibitory molecule that is sequestered and marked for proteasomal degradation upon activation of its inhibitor, inhibitor of κB kinase β (IKKβ), NF-κB is translocated from the cytosol into the nucleus where it binds in the promoter regions of the genes expressing a κB binding site [65]. NF-κB binding in such promoter regions upregulates the expression of cytokines, chemokines, and cell adhesion molecules that are involved in inflammation, augmenting the initiating inflammatory response [30,34]. This pathway is an essential component among pro-inflammatory processes, and as such, it has been explored in terms of its implications in many inflammatory diseases, including PD (Figure 5). Studies have shown that activation of the NF-κB signaling pathway is essential when it comes to the induction of apoptosis for dopaminergic neuron degeneration, a prominent clinical hallmark of PD [17]. In addition to this, NF-κB has been shown to promote the survival of neural microglia, which behave as primary mediators of chronic neuroinflammation in PD cases [30,65]. NF-κB has proven to be strongly involved in the propagation of neuroinflammatory processes in PD, feeding into one of the most pathologic components in this condition; thus, targeting this NF-κB pathway by EGCG, quercetin, and kaempferol makes these flavonoids even more promising therapeutics for the treatment of PD. In various studies, a reduction in cytokine secretion and overall allergic inflammation was achieved upon administration of flavonols like quercetin and kaempferol via attenuation of the NF-κB pathway [34]. It has also been proposed that quercetin can downregulate the NF-κB pathway by augmenting levels of the silent information regulator-1 (SIRT-1), an HDAC that has been found to be depleted among older patients with neurodegenerative diseases [42,43]. Studies have shown that by deacetylating the NF-κB complex in its RelA/p65 component, the SIRT-1 enzyme disables the transactivation capacity of the transcription factor and enhances the proteasomal degradation of this essential subunit, thereby inducing anti-inflammatory effects via the downregulation of this pathway [66]. The mechanisms through which kaempferol can attenuate this inflammatory pathway have also been further explored, with a study utilizing an in vitro GVB (gut vascular barrier) model of rat intestinal microvascular endothelial cells showing that administration of this flavonol inhibited the translocation of NF-κB into the nucleus [67]. In addition to this, EGCG has been shown to disrupt NF-κB interactions in target gene promoter sites, inhibiting the expression of these pro-inflammatory genes [13]. EGCG, quercetin, and kaempferol have all been shown to behave as effective attenuators of this strong inflammatory pathway; thus, further exploration into the molecular mechanistic abilities of these specific flavonoids ought to be conducted to gain an even better understanding of their anti-inflammatory capabilities and with it their true potential as future PD therapeutics in clinics.

## 8. Antioxidant Effects of EGCG, Quercetin, and Kaempferol

**Figure 6:**
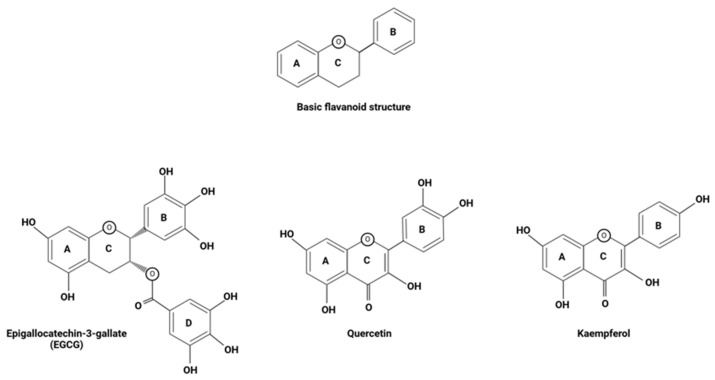
The basic structure of flavonoids, along with the compositional variations seen in EGCG, quercetin, and kaempferol. This figure was created using BioRender.

As mentioned previously, the antioxidant properties that are exhibited by flavonoids are foundational when it comes to the beneficial effects these polyphenolic compounds have been shown to induce within the body. Flavonoids behave as potent antioxidants, and studies have shown that a great deal of this property is inherent to their structure, with the amplitude of phenolic hydrogens present among this class of organic compounds not only rendering them natural, free-radical scavengers but also powerful chelating agents [34]. Chelating agents are compounds that possess electron donor groups with a high affinity for certain metals. Via covalent bonding, these structures react with transition metals such as iron to form stable chelates, sequestering the element in a non-reactive form [68]. Flavonoids have been shown to behave as natural phytochelators and thus potentially induce their antioxidant properties within the body through the chelation of free iron. There is no pathway through which excess iron can be excreted, and in its free form, this element induces toxic effects through the stimulation of free-radical formation, especially in individuals with PD who have been shown to accumulate iron in the substantia nigra [35]. Having said that, studies have shown that the sequestration of transition metals essentially ‘neutralizes’ these reactive ions, inhibiting their ability to form free-radical species and induce cellular damage, with oxidative stress levels being shown to increase in instances where sequestration activity fell [34]. Thus, there is evidence supporting the idea that the chelating abilities of flavonoids greatly contribute to their antioxidant capabilities. This further validates the potential implementation of EGCG, quercetin, and kaempferol in the treatment of PD, for studies suggest that their structures render them potent chelating agents, with the chelation of metal iron generally requiring 3- or 5- hydroxy groups, and B ring catechol groups as well as 2,3 double bonds serving as flavonoid iron binding sites (Figure 6) [35]. The antioxidant capabilities that are naturally ingrained in the chemical structure of these flavonoids immediately render them intriguing therapeutic agents when it comes to ameliorating the oxidative stress seen in PD. However, a better understanding of other potential pathways through which the intrinsic capabilities of EGCG, quercetin, and kaempferol can be augmented will lend further support for their use as therapeutic agents in the treatment of PD.

### A Nrf2 Pathway Upregulated by EGCG, Quercetin, and Kaempferol for Augmenting Their Antioxidant Capacity

**Figure 7:**
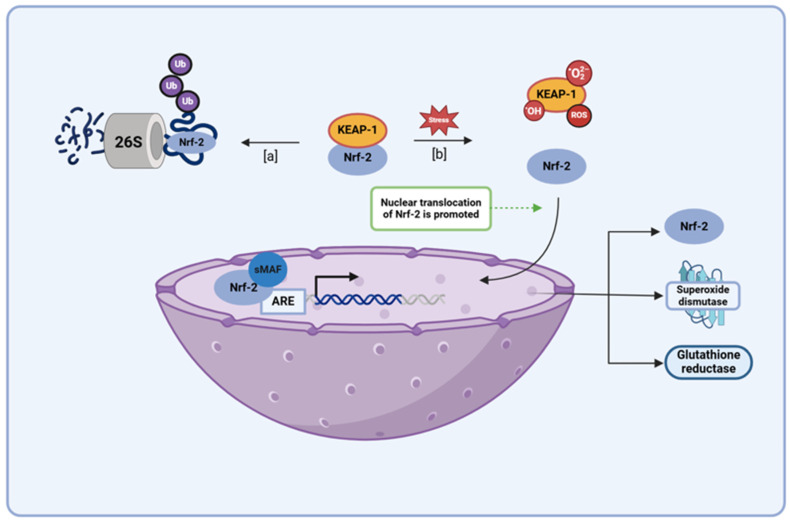
A schematic diagram depicting the KEAP1/Nrf2 pathway illustrates both the regulation of Nrf2 levels during standard housekeeping periods and the accumulation of this protein within the nucleus during times of elevated oxidative stress. (a) When oxidative stress levels are low, the KEAP1 protein maintains low basal Nrf2 levels via constitutive proteasomal degradation of the protein. (b) However, during periods of high oxidative stress, electrophiles and ROS react with KEAP1 sensor cysteines, sequestering the KEAP1 protein away from cytoplasmic Nrf2 and enabling subsequent translocation of Nrf2 protein into the nucleus. There, the Nrf2 protein binds to the ARE in the promoter region of several cell defense genes (e.g., antioxidant genes, metabolic genes, etc.), upregulating their expression during times of increased oxidative stress. The binding of small Maf (sMaf) transcription factors to Nrf2 at the protein’s leucine zipper domain aids in ARE recognition. Abbreviations: KEAP1, Kelch-like ECH-associated protein 1; Nrf2, nuclear factor erythroid 2-related factor 2; ARE, antioxidant response element; NFE2L2, nuclear factor erythroid-2 like 2. This figure was created using BioRender.

Nuclear erythroid 2-related factor 2 (Nrf2) behaves as a key transcription factor in several antioxidant pathways, with its binding in the promotor region of antioxidant response element (ARE) genes regulating the expression of several proteins involved in endogenous antioxidant mechanisms (e.g., superoxide dismutase, heme oxygenase 1, and glutathione reductase) [30,69,70,71]. Various studies have shown that therapeutic activation of Nrf2 pathways is a key mechanism for promoting antioxidant genes and preventing neuroinflammation in major CNS diseases, including PD [72,73,74,75]. During periods of low oxidative stress, Nrf2 activity is regulated by Kelch-like ECH-associated protein 1 (KEAP 1), a redox sensor that marks proteins for proteasomal degradation via ubiquitination (Figure 7) [30,76,77,78]. However, when oxidative stress levels begin to rise, ROS, electrophiles, and other cellular components displaying the KEAP1 interacting region stabilize Nrf2 content by sequestering away the KEAP 1 protein, allowing for the transcription factor to persist and undergo nuclear translocation. In the nucleus, Nrf2 forms a complex with the musculoaponeurotic fibrosarcoma (Maf) protein and binds in the promoter region of ARE genes, promoting the expression of enzymes involved in the production, utilization, and regeneration of glutathione, as well as several other endogenous antioxidants [30,76,77,78]. In addition to this, Nrf2 has been found to activate antioxidants and free-radical scavengers, adding to the functionality of this transcription factor when it comes to the role it plays within the body as an antioxidant mediator [30]. Several studies have demonstrated the therapeutic effectiveness of EGCG, quercetin, and kaempferol, and their potential application in PD treatment has shown that these flavonoids augment their own antioxidant capacity by upregulating the Nrf2 pathway. Very similar to its attenuation of the NF-κB pathway, quercetin augments the SIRT-1 level that serves as a potential mechanism through which administration of this flavonol can potentially alter the Nrf2 pathway, for several studies have suggested that the SIRT-1 may induce its antioxidant effects via the modulation of the KEAP-1/Nrf2/ARE axis [42,79,80]. In addition to this, the modulation of the Nrf2 pathway has been shown to be an essential mechanism of action when it comes to the antioxidant capabilities of EGCG and kaempferol, for studies have shown that administration of an Nrf2 inhibitor blocks the protective effects induced upon the administration of these flavonoids amidst in vivo and in vitro models of high oxidative stress [81,82,83]. EGCG, quercetin, and kaempferol have all proven to be capable of augmenting the Nrf2 pathway, with this property proving to be an essential component among a few of these flavonoids’ antioxidant capabilities; thus, their use in PD serves as a promising means through which oxidative stress levels can be attenuated through multiple potent mechanisms.

## 9. Anti-Amyloidogenic Effects of EGCG, Quercetin, and Kaempferol

EGCG, quercetin, and kaempferol have been shown to induce anti-amyloidogenic effects within the body through both the inhibition of aggregate formation and the promotion of their degradation through autophagy pathways [28,30,42]. In an in vitro study of goat brain cystatin (GBC), a dimeric protein that is predisposed to aggregation, catechin and kaempferol were found to exhibit potential inhibitory and disaggregating properties in fibril formation. The thioflavin T (ThT, amyloid reporter) study conducted in this experiment showed that co-incubation of the protein in an aqueous environment in which catechin or kaempferol were present at various doses resulted in a lower ThT emission than that seen among the samples that underwent solitary incubation, with a >50% drop in ThT emission occurring when a 1:1 molar ratio concentration was implemented and a marked reduction (>70%) occurring when flavonoid dose concentrations exceeded 50 μM. This served as an indicator that the polyphenols applied inhibited aggregation in a dose-dependent manner, and this observation was further supported using the 8-anilino-1-naphthalenesulfonic acid (ANS), an extrinsic fluorescent probe widely employed to typify proteins in various states, fluorescence study. Using the fluorescent probe that exhibited a high affinity for hydrophobic binding, this study showed that GBC proteins that underwent solitary incubation were present in a conformation that potentially supports the formation of aggregates, with several more solvent-exposed hydrophobic regions being seen upon the application of fluorescent dye than that which was seen in co-incubated GBC proteins. This indicated that both catechins and kaempferol deterred aggregation by limiting the availability of hydrophobic regions for fibril formation to happen [39]. In a separate investigation, the anti-amyloidogenic properties of quercetin, kaempferol, galangin, morin, and myricetin were explored, with an emphasis being placed on understanding what role these flavonoids might hold in the promotion of the α-synuclein protein in less pathologic pathways [61]. Misfolding and oligomerization among α-synuclein proteins have been shown to progress in one of the three distinct pathways: (a) the non-specific amorphous route in which unfolded coil monomers coalesce into small, unstructured, amorphous, and non-toxic aggregates; (b) the specific route in which unfolded coil monomers coalesce into monomeric aggregate prone intermediates (APS) that give way to structured and stable amyloid fibrils (toxic) via the infinite polymerization of formed protofibrils; (c) the specific route in which aggregate-prone intermediates undergo finite polymerization, forming non-toxic oligomeric aggregates. Through a molecular dynamic simulation of APS and α-synuclein fibrils in an aqueous environment, it was found that in the presence of flavonoids (1:10 molar ratio of α-synuclein/flavonoid), the beta-sheet content among both α-synuclein fibrils and APS structures diminished relative to the control results, while the percentages of the coil and turn content increased, respectively. Disturbance of the beta-sheet motif among fibril aggregates destabilized the toxic structure, promoting the non-specific amorphous route and giving rise to small, unstructured aggregates. This served as an indication that the flavonoids studied exhibited a high propensity when it came to altering the structural conformation of the α-synuclein protein in both its toxic fibril form and intermediate APS form, thus having the potential to both destabilize the formed aggregates as well as prevent their formation in the first place. In addition to this, assessment of the flavonoid-protein interactions taking place in the simulation indicated that the anti-amyloidogenic effects of kaempferol were highly dependent on hydrophobic interactions, while that of quercetin appeared to be more dependent on electrostatic interactions; thus, the use of these flavonoids in a combination treatment may augment their anti-amyloidogenic effects due to their unique binding properties [61]. EGCG, quercetin, and kaempferol have all been shown to exhibit anti-amyloidogenic properties; thus, their employment within PD treatment may result in the attenuation of α-synuclein aggregation within the body and the pathologic effects induced by their presence.

## 10. Critical Considerations for Enhancing the Therapeutic Efficacy of Flavonoids in PD

There is a significant amount of scientific support behind the efficacy of specific flavonoids, with several in vitro and in vivo studies providing strong evidence that they hold many health benefits in the treatment of PD; however, the real-world effectiveness of these flavonoids in the treatment of PD must be explored further, for scientific efficacy does not always translate into clinical practice.

### 10.1. Bioavailability of Flavonoids

Although research has shown that the administration of specific flavonoids has the potential to induce an array of health benefits, their bioavailability within the body hinders their use as therapeutic agents. Generally, the bioavailability of flavonoids tends to be rather low, with their high molecular weight, low water solubility, rapid elimination, food–matrix interactions, and low stability within the GI tract all serving as contributing factors to this issue [13,76]. Due to their large size, flavonoids are poorly absorbed in the small intestine, with a small percentage of the consumed content making its way into systemic circulation intact, and even among flavonoid subclasses that exhibit rapid absorption (e.g., anthocyanins), bioavailability still tends to be low, for often they are excreted as quickly as they are absorbed [28,30]. Where metabolization of these polyphenolic compounds among the liver and gut microbiota aid in the formation of easily absorbed secondary metabolites that often exhibit some level of bioactivity and long half-lives (Table 4), the process of Phase I and II metabolism is so extensive that less than 5% of polyphenolic intake enters circulation unchanged; thus, the modifications that take place within the GI system largely contribute to the low bioavailability seen among these polyphenolic compounds [30,33,40]. Attempts at overcoming this issue of low bioavailability by increasing the initial intake of flavonoids have been conducted. However, this method proved to be ineffective in cases of EGCG and quercetin, for at therapeutically effective doses both flavonoids were shown to induce negative side effects. Studies showed that the therapeutic dose for EGCG often nears or exceeds toxic levels, with negative side effects (e.g., acute liver inflammation, elevated transaminase levels, etc.) occasionally arising before any beneficial effects were seen, while administration of quercetin at high doses over an extended period of time resulted in elevated levels of quinine formation, which proved to be toxic in situations where levels of GSH were low, which happened to be the case in PD [28]. This being said, studies have shown that while acute consumption of flavonoids does not often elicit the therapeutic effects associated with these compounds due to their low bioavailability, long-term adherence to a regular intake of a diverse range of flavonoids has been shown to produce certain positive effects, with the utilization of multiple flavonoids enhancing their combined efficacy in a synergistic manner [84,85,86,87]. In addition to this, many different approaches are currently being studied to improve flavonoid bioavailability, with the use of loaded nanoparticles and the development of flavonoid pro-drugs serving as two promising pathways that are currently being explored [28,34]. Further research into the topic of bioavailability is very much needed, and particular attention ought to be paid to developing delivery mechanisms that improve upon flavonoid bioavailability without curtailing their maximal therapeutic potentials, for although metabolite production within the GI system does greatly hinder the bioavailability of the parent compound, the effects induced by these metabolites have been shown to be quite potent [28].

### 10.2. Compatibility of Flavonoids with Current Pharmaceutical Drugs in PD

Although flavonoids may prove to one day be very powerful therapeutic agents for the treatment of PD, their efficacy does not invalidate the therapeutic effects current treatments have been shown to have when it comes to symptom management; thus, exploring the manners in which flavonoids may interact with currently used PD drugs is an essential part of fully understanding their real-world efficacy. It has been suggested that due to their structural similarities to L-Dopa, catechins may act as competitive inhibitors among the catechol-O-methyltransferase (COMT) pathway and thus attenuate the peripheral metabolism of the PD drug. Through this mechanism, catechins such as EGCG may improve the bioavailability and tolerability of this major PD drug, and studies supporting this theory have been conducted. In a study of six New Zealand rabbits (male, 2.5–4.0 kg; 1–2 years old), it was found that the co-administration of L-Dopa/carbidopa with varying doses of catechin improved L-Dopa bioavailability, as indicated by improvements in the maximum concentration achieved as well as AUC_0–t_, AUC_0–∞_, and half-life values seen [91]. In a separate study, co-administration of L-Dopa and carbidopa with 150 mg/kg of tea essence dissolved in deionized water (green tea essence contains catechins, including epicatechin, epigallocatechin, epicatechin gallate, and EGCG) was shown to significantly reduce the oral clearance of the drug as well as the metabolic ratio seen, providing support that catechin reduces 3-O-methyldopa (3-OMD) formation, though the overall bioavailability of the drug was not significantly impacted (high inter rabbit variability) [80]. Additional studies ought to be conducted to further validate this potential synergistic effect and explore other potential drug interactions, implementing different drugs and flavonoids to examine if similar effects can be seen in other combinations.

## 11. Conclusions and Future Directions

PD is a neurodegenerative condition that is primarily characterized by the degradation of the dopaminergic neurons within the SN of the midbrain, as well as the motor dysfunctions that are induced by neurodegeneration. Furthermore, studies in preclinical models and with the use of clinical samples in our and other laboratories indicate that the neuropathology of PD also involves extranigral neurodegeneration [92,93,94,95,96]. PD is increasingly considered not only a brain disorder but also a multisystem disorder. As such, a multi-active therapeutic agent or a combination of multiple therapeutic agents may be required for the prevention of its pathogenesis. The gut is colloquially called the second brain in the body, and recent recognition of the existence of GBA strongly suggests that PD is most probably initiated by gut dysbiosis [97]. Flavonoids are now confirmed to be multi-active natural compounds that influence the composition and functional capabilities of the gut microbiota, and resulting microbial metabolites of flavonoids can potentially prevent α-synuclein aggregation [98,99]. Because traditional treatments for PD are essentially confined to symptom management and its prevalence has increased over the past few years, a new emphasis has been placed on discovering new therapeutic agents that can directly attenuate the causal factors of pathology involving gut dysbiosis and deter the progression of PD. Understanding gut dysbiosis and how it is potentially related to the development and progression of PD led researchers to start looking among flavonoids for a new therapeutic approach [100]. Flavonoid administration served as a means through which a dual-impact effect could be induced in PD treatment, for these macromolecules are able to both help re-establish a healthy gut microbiota and attenuate multiple molecular pathways implicated in PD, working both directly and indirectly to treat this condition. A combination of flavonoids or a combination of a flavonoid (e.g., EGCG) and L-Dopa can be a promising therapeutic strategy for the prevention of gut dysbiosis and neurodegeneration in PD preclinical models [101,102]. Artificial intelligence is expected soon to enhance PD treatments through the use of combination of specific flavonoids, combination of a specific favonoid with an existing PD drug, or combination of a specific favonoid with a repurposable drug for PD that can meet the criteria for new clinical trials for the amelioration of gut dysbiosis and multiple molecular mechanisms of PD pathogenesis [103,104,105].

This review article focused specifically on EGCG, quercetin, and kaempferol, highlighting the vast array of mechanisms that exist through which these polyphenolic compounds have been shown to prevent gut dysbiosis and attenuate the pathologic factors seen in neurodegenerative diseases, including PD. Research into their capabilities showed that EGCG, quercetin, and kaempferol can ameliorate several of the proposed etiologic factors implicated in PD through varying molecular mechanisms and pathways; therefore, their implementation serves as a potential approach through which PD can be intercepted at multiple points, dampening the cycle of pathology that is seen in this condition. The employment of multiple flavonoids also means that these pathways can be shut down through various mechanisms, amplifying the multi-hit effects of flavonoid administration; thus, combined therapeutic approaches that implement more than one of these flavonoids at a time may have the potential to induce synergistic effects in PD treatment. Both preclinical and clinical studies have shown that these compounds have the potential to act as effective natural therapeutic agents in PD; thus, further research ought to be conducted on improving bioavailability and exploring flavonoid–drug interactions to ensure this potential reaches a higher level.

## Figures and Tables

**Table 1:** Functions of the gut microbiota across varying systems in the human body and the specific bacteria and bacterial metabolites involved in the process.

Gut Microbiota-Regulated Systems	Functions	Bacteria and Metabolites Mediating Function	References
Digestive system	Bile acid transformation	*Bacteroides* spp., *Eubacterium* spp., and *Clostridium* spp.	[19]
	Tight junction regulation	Short-chain fatty acids (SCFAs)	[19]
	Mucus layer properties	*Bacteroides thetaiotaomicron*	[21]
Endocrine system	Production of neurotransmitters such as gamma-aminobutyric acid (GABA), norepinephrine, histamine, and serotonin	*Lactobacillus* spp., *Bifidobacterium* spp., *Bacillus* spp., *Escherichia* spp., *Enterococcus* spp., and *Lactococcus Lactis*	[19]
Immune system	Innate and adaptive immunity activation	SCFAs, *Clostridium* spp., and *Bacillus fragilis*	[19,21]
	Induce synthesis of antimicrobial proteins (AMPs)	*Bacteroides thetaiotaomicron* and *Lactobacillus innocua*	[21]
Nervous system	Blood-brain-barrier (BBB) regulation	SCFAs	[19]
Multiple systems	Digestion of host-resistant carbohydrates	*Bacteroidetes* spp. and *Firmicutes* spp.	[19]
	Digestion of undigested proteins and amino acids	*Clostridium* spp., *Bacteroides* spp., and *Lactobacillus* spp.	[19]
	Activation of dietary polyphenols	*Gordonibacter* spp.	[19,21]
	SCFA production	*Bacteroides* spp., *Roseburia* spp., *Bifidobacterium* spp., *Faecalibacterium* spp., and *Enterobacteria* spp.	[19,21]
	Xenobiotic and drug metabolism	*Eggerthella lenta*	[21]
	Vitamin and amino acid biosynthesis	*Bacteroides* spp., *Bifidobacterium* spp., and *Enterococcus* spp.	[19,22]

**Table 2:** Major flavonoid subclasses, their common dietary sources, proposed health benefits, and examples of flavonoids that fall within each subclass.

Major Flavonoid Subclasses	Dietary Sources	Proposed Health Benefits	Examples of Flavonoids	References
Flavones	Dried tea leaves, herbs, citrus fruit, grains, wine, olives, onions, red bell peppers, and honey	Antioxidant effects, anti-inflammatory effects, and cholesterol-lowering effects	Vitexin, apigenin, luteolin, and tangeritin	[28,30,36]
Flavanones	Citrus fruits and medicinal plants	Free-radical scavenging, antioxidant effects, anti-inflammatory effects, blood-lipid lowering, and cholesterol-lowering effects	Hesperetin, naringin, naringenin, eriodictyol, and hesperidin	[28,36]
Flavonols	Onions, green vegetables, apples, grapes, berries, tea, and certain medical plants and herbs	Anti-inflammatory effects, neuroprotection, antioxidant effects, and anti-hypertensive effects	Quercetin, kaempferol, myricetin, and fisetin	[28,36,38]
Isoflavones	Soybeans and other legumes	Phyto-estrogen effects, antioxidant effects, positive modulation of the gut microbiota, and anti-inflammatory effects	Genistein, daidzein, genistin, glycitein, and biochanin	[28,36,38]
Flavan-3-ols	Grapes, berries, pome fruits, green tea, black tea, apples, persimmons, cocoa, bananas, peaches, and pears	Improved endothelial function, improved cognitive function, antidiabetic effects, and anti-hypertensive effects	Catechins	[28,36,38]
Anthocyanin	Colorful (e.g., red, violet, and purple) leafy and root vegetables, berries, black currants, red grapes, and merlot grapes	Neuroprotection, improved vascular health, anti-inflammatory effects, improved gut barrier function, improved cognitive function, antioxidant effects, reduced excitotoxicity, and positive modulators of the gut microbiota	Pelargonidin, cyanidin, delphinidin, peonidin, petunidin, and malvidin	[28,36,38]

**Table 3:** Recent clinical trials that emphasize the importance of mitigating mitochondrial dysfunction and oxidative stress in PD.

Identification Number	Study Design	Study Description	Results	Study Timeframe
NCT04300608	Cross -sectional cohort study	30 elderly individuals (65–85 yo) with early to mid-stage PD (Hoehn and Yahr score 2–3) were evaluated based on the metabolic and functional properties of their skeletal muscle, and the findings of these assessments were then compared to a control dataset of healthy individuals who fell within the same age demographic.	NA	November 2021–Unknown
NCT00329056	Double-blind, prospective, randomized control study (Phase 2 trial)	128 individuals with untreated PD were randomly assigned to one of three different clinical arms, consisting of two experimental arms (40 mg Mito Q tablets and 80 mg Mito Q tablets) and a control arm (placebo group). Then, over a 1 year period, the individuals in this study were assessed on the progression of their condition using the UPDRS and evaluated for any pernicious effects.	NA	May 2006–November 2007
NCT03421899	Prospective observational study	160 individuals with PD or of good health were stratified into three groups: individuals with monogenic forms of PD that involved some form of mitochondrial dysfunction, individuals with idiopathic PD, and healthy individuals. Throughout a three-year period, DNA-containing samples were collected and subjected to biochemical assays in the hopes of discerning if there were any biochemical markers that could be used to differentiate individuals with PD based on mitochondrial dysfunction.	NA	August 2017–April 2020
NCT03061513	Quadruple-blind, randomized control study	11 individuals who were diagnosed with PD within 5 years of study participation were randomly assigned either to the experimental arm in which 600 mg of ubiquinol were administered daily over 24 weeks or the control arm in which a placebo was administered daily over the same time frame. Throughout the study period, participants were assessed for any adverse effects and cerebral redox markers, with an MRI examination being integrated into the study at baseline and again at the end of 8 weeks of treatment.	Number of adverse effects: Experimental: 27 Control: 12 Number of serious adverse effects: Experimental: 0 Control: 0 Change in cerebral redox markers–lactate levels [Mean (Standard deviation)]: Experimental: ∆ − 11.98 (18.57) Control: ∆ 6.23 (23.19)	28 February 2012–31 December 2017
NCT02462603	Within-subject, controlled open-label study (Phase 2A trial)	44 individuals who were diagnosed with late-stage idiopathic or monogenic PD received a dose of 500 mg PTC589 orally twice daily for up to 3 months unless the administration must be discontinued due to safety concerns. Throughout the treatment phase, participants were assessed for peripheral blood biomarkers, CNS biomarkers, urine biomarkers, disease progression, and side effects.	Severe adverse effects (41 participants): 0 Change in disease severity from baseline (increasing score indicates worsening severity; 40 participants): nM-EDL: ∆ − 0.1 (3.93) M-EDL: ∆ 0.1 (3.54) Motor examination: ∆ − 0.6 (6.72) Motor complications: ∆ − 0.1 (2.17) Change in non-motor symptoms scale scoring (40 participants): ∆ − 0.6 (14.79) (Further outcome measures were discussed in the results but were excluded here for brevities sake)	17 May 2016–08 January 2019

yo = years old; NA = not available; UPDRS = unified Parkinson’s disease rating scale; MRI = magnetic resonance imaging; nM-EDL = non-motor experiences of daily living; M-EDL = moto experiences of daily living.

**Table 4:** The pharmacokinetics and overall bioavailability of flavonoids.

Study Design	Administration	Pharmacokinetics	Reference
Single arm study Subjects: 10 healthy men; 21–28 years old Duration: 48-h intervention study; single dose administration; sample collection (blood and urine) occurred throughout the intervention period	6.3 mL/kg body weight of ‘juice mix’ containing quercetin (30 mg/L juice mix), naringenin (28 mg/L juice mix), and hesperetin (32 mg/L juice mix)	Quercetin T_max_ plasma (h): 3.6 ± 1.6 C_max_ plasma (μmol/L): 0.15 ± 0.13 AUC_0–48 h_ (μmol × h/L): 1.77 ± 1.63 Mean excreted amount 0–48 h (μg): 227 ± 142 Accumulated relative urinary excretion 0–48 h (% of the dose): 1.5 ± 1.0 Naringenin T_max_ plasma (h): 3.6 ± 1.6 C_max_ plasma (μmol/L): 0.25 ± 0.13 AUC_0–48 h_ (μmol × h/L): 2.82 ± 2.09 Mean excreted amount 0–48 h (μg): 3160 ± 1612 Accumulated relative urinary excretion 0–48 h (% of the dose): 22.6 ± 11.5 Hesperetin T_max_ plasma (h): 4.9 ± 1.4 C_max_ plasma (μmol/L): 0.18 ± 0.13 AUC_0–48 h_ (μmol × h/L): 1.99 ± 1.49 Mean excreted amount 0–48 h (μg): 2278 ± 1457 Accumulated relative urinary excretion 0–48 h (% of the dose): 14.2 ± 9.1	[37]
A double bind randomized cross-over study Subjects: 15 participants: 12 males and three females; 22–55 years old; BMI 18–25 kg/m^2^ Duration: a preliminary low fisetin diet was adhered to for a 2-day period prior to the start of the study; single dose administration; sample collection (blood) occurred over a 12-h period	Capsule (1000 mg) of unformulated fisetin (98.2% fisetin content; oral administration)	C_max_ (ng/mL): 9.97 ± 3.97 T_max_ (h): 0.88 ± 0.18 T_1/2_ (h): 1.14 ± 0.09 AUC_0–12_ (ng × h/mL): 12.67 ± 4.86	[88]
A randomized six-sequence/three-period cross-over study (the researchers were aware of which treatment was administered among participants, but did not know which treatment was associated with a sample once it was collected) Subjects: 12 healthy participants; 18–50 years old; BMI 18.5–27 kg/m^2^ Duration: a low quercetin diet was adhered to for a period of at least 72 h prior to the start of the study; single-dose administration; sample collection (blood) occurred over a 24-h period	Solubility study: 20 mg of quercetin dissolved in 10 mL of a stimulated biological fluid Pharmacokinetic study: 500 mg film-coated quercetin tablet (oral administration)	Solubility: FaSSGF pH 1.6 ≤ LOD FaSSIF pH 6.5–0.0075 mg/mL FeSSIF pH 5.0–0.0191 mg/mL C_max_ (ng/mL): 10.93 ± 2.22 T_max_ (min): 290.00 ± 31.19 T_1/2_ (min): 375.63 ± 75.51 AUC_last_ (min × ng/mL): 4774.93 ± 1190.61	[89]
Single arm study Subjects: six participants; 18–65 years old; randomly sampled from a larger clinical trial focused on studying the cardioprotective effects of Aronia berry supplementation among former smokers at risk for cardiovascular disease; BMI 18.5–39 kg/m^2^ Duration: a low polyphenol diet was adhered to for at least 3 days before the study began, followed by an overnight fast; single dose administration; sample collection (blood and urine) occurred over a 24-h period	Approximately 500 mg of Aronia extract (two 250 mg extract capsules taken with water) consisting of: Cyanidin-3-galactoside + cyanidin-3-glucoside: 32.52 ± 0.7 mg Cyanidin-3-arabinoside: 11.79 ± 0.3 mg Cyanidine-3-xyloside: 0.76 ± 0.0 mg [Total: 45.1 mg anthocyanins]	Cyanidin-3-galactoside Plasma C_max_ (ng/mL): <LOD Plasma T_max_ (h): N/A Plasma AUC (μg × h/mL): N/A Urine C_max_ (mg/mg creatinine): 0.004 ± 0.001 (0.002–0.010) Urine T_max_ (h): 4.67 ± 1.03 Urine AUC (μg × h/mL): 0.016 ± 0.005 Cyanidin-3-glucoside Plasma C_max_ (ng/mL): 0.059 ± 0.024 (0.014–0.180) Plasma T_max_ (h): 1.60 ± 0.24 Plasma AUC (μg × h/mL): 0.462 ± 0.170 Urine C_max_ (mg/mg creatinine): 0.010 ± 0.006 (0.002–0.004) Urine T_max_ (h): 6.00 ± 3.35 Urine AUC (μg × h/mL): 0.118 ± 0.070 Cyanidine-3-arabinoside Plasma C_max_ (ng/mL): <LOD Plasma T_max_ (h): N/A Plasma AUC (μg × h/mL): N/A Urine C_max_ (mg/mg creatinine): 0.020 ± 0.006 (0.002–0.038) Urine T_max_ (h): 4.00 ± 1.26 Urine AUC (μg × h/mL): 0.088 ± 0.031	[90]

C_max_ = maximum plasma concentrations; T_max_ = time taken to reach maximum plasma concentration; t_1/2_ = half-life in plasma circulation; AUC = area under the plasma concentration vs. time curve; FaSSGF = fasted state simulated gastric fluid; FaSSIF = fasted state simulated intestinal fluid; FeSSIF = fed state simulated intestinal fluid; LOD = limit of detection.
